# Laboratory automation —some perspectives on the challenges in the implementation of the technology in pharmaceutical development

**DOI:** 10.1155/S1463924698000169

**Published:** 1998

**Authors:** Nigel North, Simon Smith

**Affiliations:** 1 Pharmaceutical Technologies SmithKline Beecham Pharmaceuticals Essex Harlow UK; 2 Analytical Development SmithKline Beecham Pharmaceuticals Sussex Crawley UK

## Abstract

The intensifying pressure on reducing the development time for new pharmaceutical products is resulting in an increasing need for laboratory automation. A key element for the successful implementation of robotics for drug product analysis is the establishment of a reliable process for interaction of the automation team with its various customers, for example development product team and manufacturing group. The reduction of cycle time for product development appears to be resulting in more stability studies to support NDA/MAA filings for several reasons. Key clinical information may not be available before initiation of the stability studies and simultaneous world-wide development may result in an increase in the number of product strength and pack options.


Journal of Automatic Chemistry, Vol. 20, No. 4 (July–August 1998) pp. 111–115

### Laboratory automation—some perspectives on the challenges in the implementation of the technology in pharmaceutical development

**Nigel North**
*Pharmaceutical Technologies, SmithKline Beecham Pharmaceuticals, Harlow, Essex, UK*

**and Simon Smith**
*Analytical Development, SmithKline Beecham Pharmaceuticals, Crawley, Sussex, UK*

*The intensifying pressure on reducing the development time for new pharmaceutical products is resulting in an increasing need for laboratory automation. A key element for the successful implementation of robotics for drug product analysis is the establishment of a reliable process for interaction of the automation team with its various customers, for example development product team and manufacturing group. The reduction of cycle time for product development appears to be resulting in more stability studies to support NDA/MAA filings for several reasons. Key clinical information may not be available before initiation of the stability studies and simultaneous world-wide development may result in an increase in the number of product strength and pack options.*

### Introduction

Pharmaceutical Technologies at SmithKline Beecham (SB) is responsible for the formulation and analytical development, clinical trials supply and technology transfer of new products to manufacturing facilities. The department is organized with a team-based structure in which the formulation and analytical development is performed by dedicated 'product' teams for a development candidate. More generalized activities, such as microbiological and raw materials' testing, are the responsibility of 'support' teams. Implementing robotics/automation is the mandate of the Analytical Technologies Development (ATD) Support team (see figure 1).

This central team works with the Product teams to implement robotic analytical methods employing the general process shown in figure 2 using the following equipment: Zymark TPW™ IIs, Zymark MultiDose™ (USP2) and Zymate™ dissolution robot (USP 1). Over the past six to 12 months improvements to the implementation of this process have been identified and are discussed below.

### Discussion

### Survey projects

Before work on automating products is initiated, periodic surveys are performed to update information on the status of projects in the development pipeline. The results of this process lead to a prioritized list of projects for the team to develop and validate methods. As a central support team there is inherent difficulty in finding a time-efficient way of keeping up to date with changes in projects that may affect work on the projects. This may include changes in dosage form strength, in formulation and in project timings as the results from the clinical trials become available. More recently, it has been found that central budgets for projects have been a good reflection of changes and this is used to keep up to date with the projects.

The destination of a product for commercial manufacture is an important consideration. Discussions are held with the manufacturing site to determine if there are any unforeseen problems in their ability to use the automated methods. In some instances particular sites may not have the appropriate equipment to take on the automated method and early notification will enable budgets to include purchase of the required equipment.

### Prioritize and plan

Once a listing of projects has been obtained, the timings for automation need to be identified. Currently, samples for NDA/MAA stability studies are being targeted for robotics. However, this timing has been found to be too late in some instances since Product teams can be too busy to adopt the automated method as resources are being used to support other activities such as the manufacture of the qualifying batches. Consequently, the goal is to automate products sooner than Phase 2B/3 in the development cycle to overcome this problem, however, this needs to be balanced against late changes in strength and formulation that can occur resulting in wasted effort on automating redundant formulations.

In addition, the collection of metrics on the key elements of the process described in figure 2 has been initiated to provide data on the effort required to implement robotic methods. This will enable a better assessment of the overall benefit of using robotic methods for 'smaller' projects. Estimation has also been made of the cost benefit to production sites of the provision of ready validated robotic methods (see table 1). In the future, it is planned that implementation of robotic methods will be rapid enough to make it cost effective to automate projects from Phase I.

0142-0453/98 $12 00 © 1998 Taylor & Francis Ltd

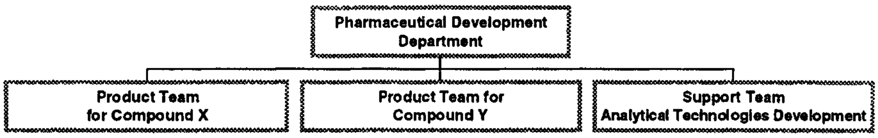

*Figure 1. Organizational structure.*

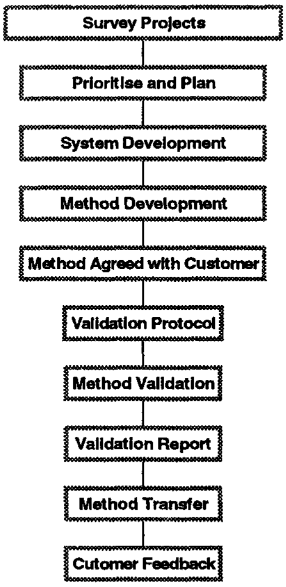

*Figure 2. Implementation process for robotic automation.*

*Table 1. Relative cost benefit of manual and automated methods.*

| Method type | Time (h) | Cost (£) |
|---|---|---|
| Manual | 22.5 (3 days) | 1350 |
| Automated | 7.5 (1 day) | 450 |

*Note*: Based on two analysts @ £30 per hour each.

*Systems development*

For the majority of the automation projects, the Zymate™ dissolution robot, Zymark Tablet TPWII™ and Zymark MultiDose™ dissolution systems are the turnkey instruments used so there is little need for system development. It is envisaged that this equipment will be routinely used by the Product teams with involvement from the Analytical Technologies Development (ATD) team only when problems occur and troubleshooting is needed. The ATD team is responsible for ensuring that the equipment used is qualified appropriately, which may involve working with the vendor of the instrumentation for turnkey systems for performing the work internally for customized equipment.

The next generation of automated systems will need to increase the speed of analysis for pharmaceutical dosage forms. First, there will be increase in development candidates generated by Discovery as a result of technologies like combinatorial chemistry. Second, there has been an increase in the number of stability studies as a result of parallel development and global commercialization of products. For example, for some indications, special packaging and dose strengths are needed by Japan. Also, in commercial operations there is an increased need for flexibility in the supply chain, which has resulted in extra stability studies to support change in manufacturing site.

The result of these trends has been that on average during development of a solid oral dosage form for a single indication; four strengths of tablet are formulated and three pack options are qualified for marketing, culminating in 150–250 stability studies being performed. Clearly this is a massive resource load that automation must help reduce. To further exacerbate this situation when stability samples are taken off store the analyses need to be completed in a specified timeframe. For one SB product, 100 dissolutions, 100 assays and 100 degradation profiles needed to be performed in 10 working days. Automated dissolution equipment is necessary to complete this task.

Reducing the turnaround time for analyses to meet the increasing workload could be achieved by developing the following technologies:

- Fibre optics for *in situ* analysis. This technology has been already been implemented by other workers [1] for dissolution procedures and could, in principle, be applied to content uniformity and assay methods.
- Systems of reduced size to facilitate on-line/at-line analysis. This is particularly important for USP1 dissolution where fully automated robotic units can take up a lot of space.
- Faster analysis by using technologies such as micro liquid chromatography and capillary electrochromatography. Also, with the development of cheaper and more user-friendly LC/MS systems, it may be feasible to follow the lead shown in the analysis of biofluids and employ this technology for rapid analysis of drugs in dosage forms particularly for highly potent low dose products. The successful use of these technologies would reduce runtimes for analysis from about 15 min down to about 3 min. As a consequence, the speed of sample preparation, typically 20 min for the Zymark TPWII™, would be the rate limiting for on-line analysis and significant improvements in this are needed.
- Improved links between robotics and LIMS systems to enable easier movement of data is critical. This is particularly important for the extra data available from robotic systems (for example sample weights, solvent volumes, dilution volumes, etc.) that are currently archived separately from the assay result information which is stored in LIMS. Ideally, these

*Table 2. Generic extraction solvents.*

| Aqueous solubility of drug (mg/ml) | Extraction solvent |
|---|---|
| > 1 | Aqueous buffer |
| 1–0.05 | Methanol/aqueous buffer |
| < 0.05 | High methanol content |

data should be available for review from the LIMS system for audit purposes.

*Method development*

In order to promote the use of robotics, it is intended to provide method development guidelines for the Zymark TPWII$^{TM}$ which should also give a better understanding of the requirements for automated methods and promote method harmonization. Currently, the extraction system for automated methods is based on that already available for the manual procedure which has resulted in some instances in significantly different extraction solvents being used for compounds from a similar class or for product line extensions of the same compound. For one major project, six different extraction solvent/conditions are used for solid oral dosage forms. The goal is to move towards preferred extraction solvent conditions for compounds depending on its solubility as shown in table 2. It is intended that these solvent conditions are employed first, if this is not successful, alternative solvents are investigated.

Manual methods typically use shaking and ultrasonication procedures for extraction of the drug from the formulation matrix. It may be appropriate to suggest investigation of homogenization as an alternative manual procedure in the method development guidelines to enable easier transfer of methods to the TPWII$^{TM}$.

Experimental design is also being used to optimize the Zymark TPWII$^{TM}$ extraction procedure. The success of this technique is dependent on real differences being observed in the results. For example, it has been found that the inherent variation in the assay of a batch of drug product can provide misleading conclusions on the significance of an extraction parameter. Increasing the replication for each set of conditions avoids this phenomenon. Also, occasional spuriously low results have been obtained and interrogation of the audit trail reveals no obvious reason can be determined leading to confounding of the results. It is suspected that incomplete break-up of tablet may be the reason, but there is no evidence to support this hypothesis. Monitoring of the extraction vessel during optimization of the extraction using a video camera could help in problem diagnosis.

The responsibility for development of robotic methods currently lies with the ATD team. As Product teams become more familiar with the technology, and as the guidelines for automated methods become available, it is intended that the responsibility for this step in the process moves towards the Product team with the ATD team acting in a consultative capacity.

The possibility of contracting out this function is also being investigated to enable faster implementation of automated methods. this will become particularly important as new technologies, like combinatorial chemistry, result in more compounds entering development which, in turn, will mean the automated method development capacity of Pharmaceutical Technologies being exceeded. It may also be appropriate to have robotic methods developed by contractors for more 'mature' products with long tedious extraction procedures (for example controlled release products).

*Method agreed with customer*

On the completion of development of the method, feedback is obtained from the Product team to ensure the procedure satisfies their needs. For MAA/NDA stability studies, the aim is to provide a suite of automated methods including assay, degradation profile and dissolution procedures. Agreement is also sought from Production to ensure, where feasible, that requirements have been met. In the commercial environment, the emphasis for automation is on the release testing of product, whereas, in Research and Development, automation of stability testing methodology is the priority. For example, in Pharmaceutical Technologies there is a general preference for HPLC methods for assay, whereas some Production sites have a preference for UV procedures for 'stable' compounds. For assay methods using the Zymark TPWII$^{TM}$ the rate limiting step is the speed of the sample preparation rather than analysis since the time for the HPLC or UV assay does not add significantly to the overall analysis time.

*Validation protocol*

Validation protocols [2] are agreed with the Product team before work is initiated, so the appropriate samples and resource can be provided. As more experience is obtained, it is anticipated the protocols will become generic with little variation from product to product.

It has been found the acceptance criteria for the comparison of the assay results from the manual and automated methods need to be carefully selected. Reliance on statistical tests has caused problems for two reasons. First, the precision of the automated procedure can be significantly better than the manual procedure. Second, the homogeneity of the batch of drug product used for showing equivalence to the manual method may be variable enough for statistical differences to be found, particularly for low dose compounds. The acceptance criterion generally used is that the mean of the manual and robotic results must agree within 2.0%. Ideally from a statistical viewpoint it is desirable to test six batches for comparison purposes, however, during the development of a product there are limited numbers of batches available to compare the automated and manual methods. A pragmatic approach has been taken ensuring at least one batch of the highest and lowest strengths of the drug product are tested for the comparison.

*Method validation*

Currently, method validation is performed by the ATD team since the robotic technology is relatively new to the department. Earlier implementation of automated procedures (for example Phase 2A), together with the possibility of seconding a member of the Product team into the ATD team to perform method validation will be investigated. Using a contract laboratory for this work will also be examined. In the long term, with the availability of validation guidelines for automated methods and increased familiarity with the equipment, it is envisaged that the Product teams will have the expertise to perform this task. This should significantly increase the rate at which new methods can be introduced.

In the UK, an inter-company pharmaceutical group is developing a common set of validation guidelines for assay/content uniformity and dissolution testing. When agreed, it is planned to publish these guidelines.

*Validation report*

The completion of the validation report for the automated method is a key deliverable that needs to be completed before the automated method can be used. Currently the onus is on the ATD team to generate this report. The validation report produced is approved by the ATD and the Product teams. It is the Product team's responsibility to incorporate the information into their existing report for the manual method. Generic templates for validation reports should be available as more methods are validated. This should speed up the rate at which the ATD team can complete this task.

*Method transfer*

In order to transfer methods successfully, training of the Product teams on the automated systems is a critical aspect of the ATD team's role. The user friendliness of the robotic systems has improved particularly with the introduction of the Benchmate™/TPWII™/MultiDose™ systems. However, unless these systems are used on a regular basis, familiarity and ease of use will be lost. It has been found that retraining of Product teams occurs too frequently. Consequently, the drive to establish automated methodology earlier in the product development process is a priority.

Transfer of automated methods to SB production facilities should be a relatively simple process, i.e. a method on a computer disk. The Production sites have invested in robotic equipment to gain not only cost savings [3] but also to reduce the method transfer time and resource. The protocol to transfer of the automated method from site to site is generally based on comparing data from at least three batches of drug product. The acceptance criterion is that the mean of the results is within 2.0%. The manufacturing sites have found robotic methods to be generally more robust than manual procedures.

In order to gain most benefit from robotics, it is important that commercial manufacturing sites have the same type of robotic equipment as that used by Research and Development. The method development and validation performed in Pharmaceutical Technologies can be used by production sites, which results in reduced time and resource in transferring methods. For major projects, more than one site is often involved in commercial manufacture, so proportionately greater savings are generated. Production sites within SB have recognized these advantages and have invested in the same robotic platforms which comprise Zymark TPWII™ and MultiDose™ systems. In order to co-ordinate automation activities between Pharmaceutical Technologies and Production, regular communication is achieved through teleconferences and, in the future, a database will be implemented to aid sharing of information. For example updates on the status projects in Research and Development and on equipment evaluations will be given.

Contract laboratories are now beginning to invest in robotics and outsourcing stability testing using automated methods is being actively investigated. It is anticipated that the same type of cost savings described above for the transfer of methods to SB Production facilities will be achieved.

*Customer feedback*

In the past, the reliability of our robotic systems has been poor, resulting in the Product team becoming frustrated with the systems and turning back to their manual methods. More recently, however, with advent of upgrades in software and purchase of newer equipment, reliability has reached a level that does not seem to affect the user significantly. Metrics are now being collected using the key steps in the process described in figure 2, including the time taken to develop and validate robotic methods, together with logging of the number of samples run on the automated equipment. This should provide information on improvements in the implementation process for automated methods and should also generate data to help justify future equipment purchases. As mentioned above, meetings have been initiated with Production to understand their needs and to obtain feedback on methods that have been transferred.

### Summary

A process for the implementation of robotics for product analysis has been defined and improvements highlighted. Major Phase 2/3 products are targeted for robotics and these methods are used for stability studies to support regulatory filings. The reduction of cycle time and worldwide product development appears to be resulting in more stability studies to support NDA/MAA filings. Implementation of technologies to reduce the analysis time have been outlined, which will result in the requirement to increase the speed of sample preparation.

Technology transfer of robotic analytical methods to manufacturing is relatively simple, providing the QC laboratories at the commercial production site make the strategic decision to align their robotic equipment with that available in Development. At present few contract testing laboratories have robotic automation available, which restricts outsourcing of large stability studies using this technology.

### Acknowledgements

Nigel North wishes to thank John Stanley, John Gostick, Les Brockhurst, Ian Gower, Chris Banton and Brian Newland for their help in developing the robotic systems in Pharmaceutical Technologies.

The authors also acknowledge Danny Evans for his managerial support of automation in Pharmaceutical Technologies.

### References

1. Kostek, L. J., 1995, in *Proceedings of International Symposium on Laboratory Automation and Robotics*, pp. 355–367.
2. Smith, S., 1996, in *Proceedings of International Symposium on Laboratory Automation and Robotics*, pp. 512–522.
3. Smith, S., 1995, in *Proceedings of International Symposium on Laboratory Automation and Robotics*, pp. 299–313.

